# Atrial Wall Perforation After Atrial Septal Defect Device Closure: A Case Report

**DOI:** 10.7759/cureus.81411

**Published:** 2025-03-29

**Authors:** Samia Bekheet, Riad Abouzahr, Zaheer Ahmad, Osama A Abdulrahman, Aly A Yousef

**Affiliations:** 1 Pediatric Cardiology, Cairo University, Cairo, EGY; 2 Pediatric Cardiology, King Faisal Specialist Hospital and Research Centre, Jeddah, SAU; 3 Cardiothoracic Surgery, Madina Cardiac Center, Madina, SAU; 4 Pediatric Critical Care, King Faisal Specialist Hospital and Research Centre, Jeddah, SAU

**Keywords:** atrial septal defect (asd), cardiac perforation, intervention pediatric cardiology, pediatric cardiac surgeries, transcatheter closure device

## Abstract

Transcatheter device closure is currently the first modality of choice in the management of secundum atrial septal defects (ASDs). We report a seven-year-old child with secundum ASD who underwent transcatheter ASD device closure using the Amplatzer septal occluder (ASO) (AGA Medical Corporation, Golden Valley, Minnesota, United States). Two weeks after the procedure, the patient presented with life-threatening acute pericardial effusion as a result of atrial roof erosion. Diagnosis was confirmed by echocardiography and cardiac CT scan. Surgical removal of the device and repair of the tear in the atrium together with ASD closure were done. Although rare, cardiac erosion is a significant and life-threatening complication after transcatheter ASD device closure that should not be underestimated.

## Introduction

Atrial septal defects (ASDs) are common congenital heart defects. They frequently coexist with additional heart abnormalities. ASDs comprise 8-10% of all congenital heart diseases with a prevalence of approximately 56 per 100,000 live births [1]. In the first year of life, 20% of ASDs will close on their own. Ostium secundum defect makes up about 80% of ASDs [2].

ASD closure can be performed by surgical sternotomy, thoracoscopic ASD closure, or the transcatheter method. The first attempt at transcatheter ASD closure was performed by King and Mills in 1976 [3]. Studies have shown that transcatheter closure of secundum ASD is less invasive and has lower complication risks and shorter duration of hospital stay compared with surgical closure [4,5].

Risks associated with transcatheter ASD closure include residual shunt, malposition and dislocation of occluders, embolization, arrhythmias, and thrombo-embolization into the central nervous system in addition to device impingement on caval veins, the right upper pulmonary vein, and the mitral and tricuspid valves [6,7].

Some studies have reported a rare complication of using ASD device closure which is related to cardiac perforation or the development of fistulas between the atria and the aorta as a result of the percutaneous ASD closure [8-14].

We report a case of atrial roof perforation two weeks after transcatheter ASD closure.

## Case presentation

A seven-year-old child with a large ASD, recurrent chest pain, and palpitation was referred to our hospital. Echocardiography demonstrated a large secundum ASD (15-16 mm) with left to right shunt and mild tricuspid regurgitation with dilated right atrium and right ventricle.

**Figure 1:**
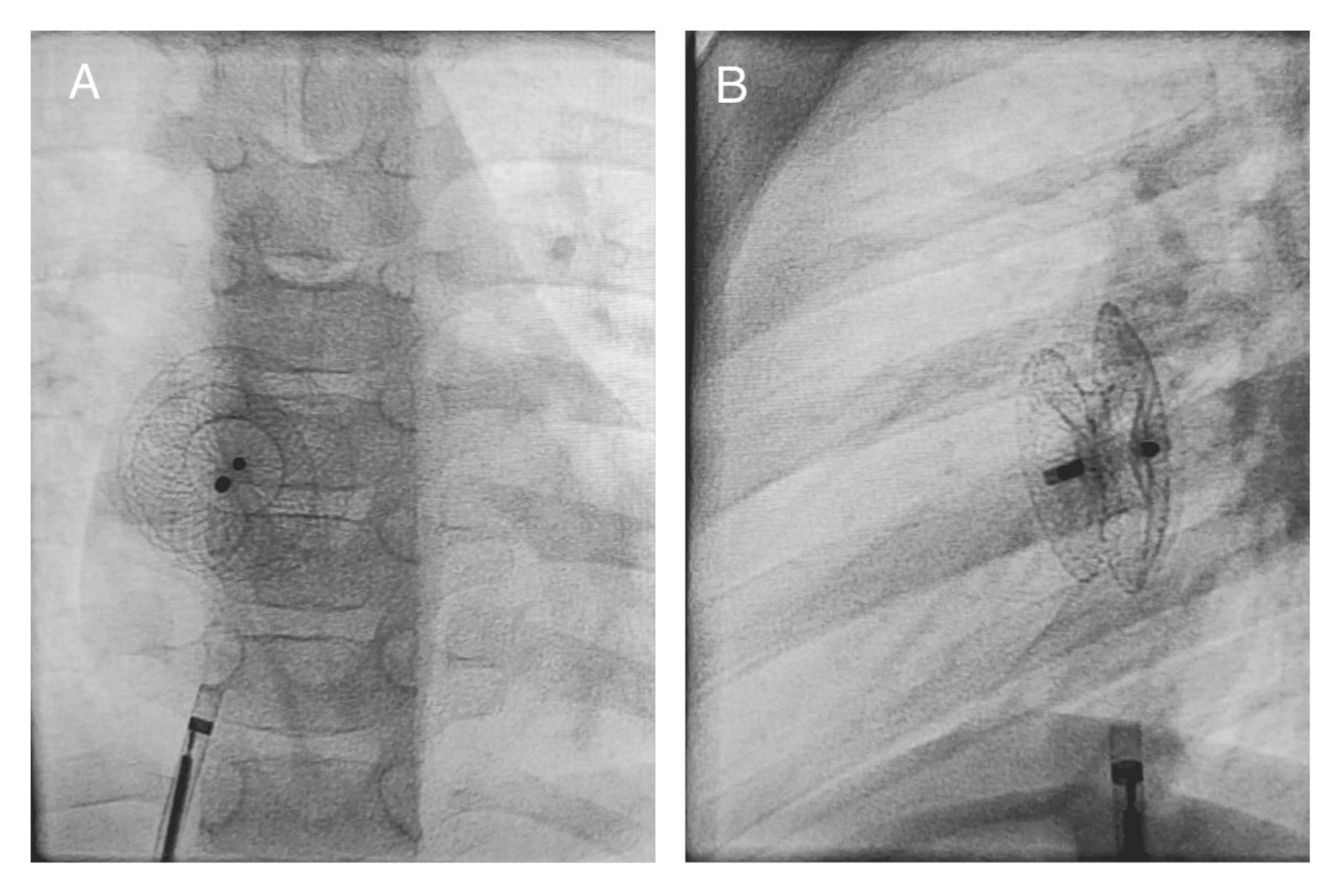
Fluoroscopy after ASO device release (A) Frontal and (B) lateral projections showing both discs of the ASD device in good place ASO: Amplatzer septal occluder; ASD: atrial septal defect

The patient was referred for transcatheter ASD device closure. The procedure was performed using a 6Fr right femoral vein sheath. An 18 mm Amplatzer septal occluder (ASO) device (AGA Medical Corporation, Golden Valley, Minnesota, United States) was used to close the defect, and its position was confirmed by fluoroscopy (Figure 1) and transthoracic echocardiography (TTE).

**Figure 2:**
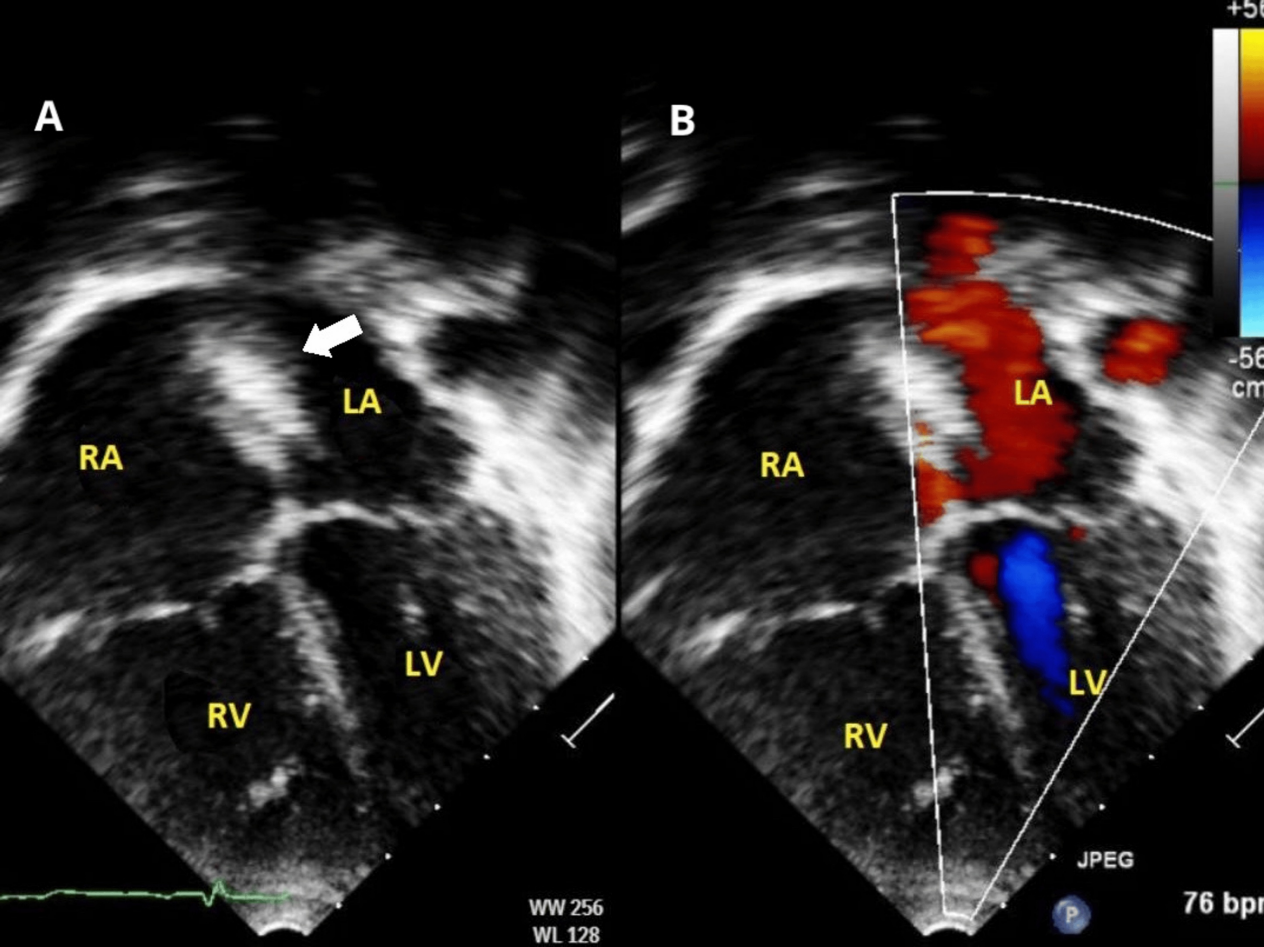
TTE 24 hours after ASD device closure (A) Apical four-chamber view demonstrating the ASD device (arrow) in place with no encroachment on the atrial wall or atrioventricular valves. (B) Colored apical four-chamber view showing no significant residual shunt LA: left atrium; LV: left ventricle; RA: right atrium; RV: right ventricle; TTE: transthoracic echocardiography; ASD: atrial septal defect

After observation for 24 hours, a predischarge TTE showed the ASD device to be well seated with no residual shunt and no encroachment on the atrial wall or atrioventricular valves (Figure 2). The patient was discharged on an antiplatelet as prophylaxis.

Two weeks later, the patient presented to the emergency department complaining of sudden chest pain followed by repeated vomiting and lethargy. On examination, he was pale, tachycardic (120/min), and hypotensive (85/55 mmHg).

**Figure 3:**
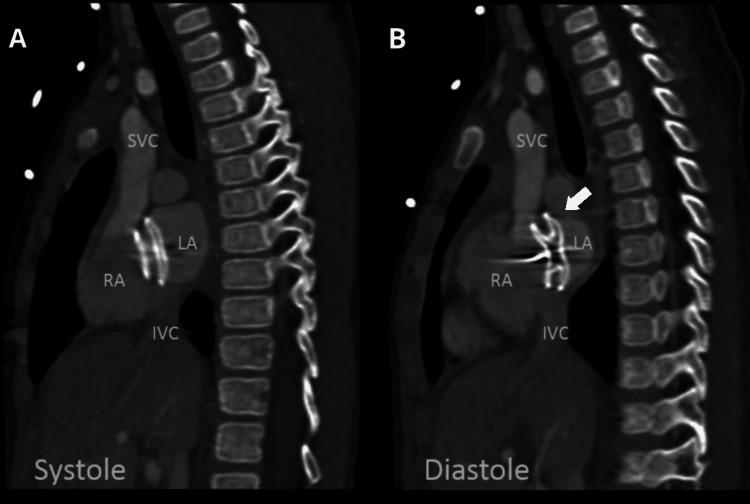
Cardiac CT angiography in the sagittal oblique (bi-caval) projection Both discs of the ASD device within the atrium in systole (A). However, in diastole (B), there is significant superior displacement of the left atrial disc into the roof of the left atrium (arrow) RA: right atrium; RV: right ventricle; LA: left atrium; SVC: superior vena cava; IVC: inferior vena cava; ASD: atrial septal defect

Urgent echocardiography revealed a significant pericardial effusion with right atrial collapse. Urgent pericardiocentesis was performed under TTE guidance, and about 115 ml of hemorrhagic fluid was aspirated. Cardiac CT scan demonstrated dynamic movement of the left atrial disc with small protrusion through the left atrial roof for a distance of 3 mm in diastole, suggestive of erosion (Figure 3).

The patient was taken to the operating room for ASD device removal. The right atrium was opened, and the device was extracted. There was a tear in the roof of the left atrium near the root of the aortic valve. The tear was repaired from both the epicardium and endocardium by direct stitch. No other tears were identified, and the ASD was closed by pericardial patch.

No postoperative complications were reported. Echocardiography showed no residual ASD or pericardial effusion, and the patient was discharged in stable condition.

## Discussion

Due to the procedure's efficiency and safety as well as the benefit of a quick learning curve, percutaneous ASD closure has become a common intervention. A serious side effect of this procedure is erosion of the atrial wall, which might result in cardiac perforation, tamponade, and death.

Early and late atrial erosions have been reported [11-14]. Most of the erosion took place close to the aortic root and the top of the atrium, and it may have affected the roofs of the left atrium, the right atrium, or both. About 87.6% of erosions occur within the first year following implantation. Around 57% of children experienced erosions within 72 hours of the procedure, while 65% of adults do so later [15].

Tissue erosion has been linked to a number of risk factors: large device size, a defective or nonexistent superior-anterior rim (considered deficient if the length is 5 mm and absent if the length <1 mm), encroachment of the device on the posterior atrial wall, under- or oversizing of the device, and device straddling of the aorta [8].

In our case, the patient presented with acute chest pain two weeks after the procedure as a complication of cardiac erosion. This case demonstrates one of the potentially fatal side effects of percutaneous ASD closure. Careful assessment of ASD size and rims by balloon sizing test in addition to the use of transesophageal echocardiography may reduce the risk of such complications. Early and rigorous lifelong follow-up is essential even in perfectly seated ASD devices.

## Conclusions

With the development of science and technology, the treatment of ASD by transcatheter device closure has become one of the most successful and modern effective cardiac interventions. Cardiac erosion is a significant complication, although rare, that should not be underestimated. Careful assessment of risk factors before the procedure and early and lifelong follow-up are indicated.
